# House price prediction using a hybrid GRU–MLP based on binary whale optimization algorithm and ant colony optimization for hyperparameter tuning

**DOI:** 10.1038/s41598-026-62854-z

**Published:** 2026-07-25

**Authors:** Sarah M. Alhammad, Yasser Fouad, Amira A. Mahmoud, Ahmed M. Osman, Hazem M. El-Bakry, Ahmed M. Elshewey

**Affiliations:** 1 Department of Computer Sciences, College of Computer and Information Sciences, Princess Nourah Bint Abdulrahman University, 11671 Riyadh, Saudi Arabia; 2 Department of Computer Science, Faculty of Computers and Information, Suez University, P.O.Box:43221, Suez, Egypt; 3 Department of Computer Science, Future Higher Institute for Specialized Technological Studies, Cairo, Egypt; 4 Department of Information Systems, Faculty of Computers and Information, Suez University, P.O.Box:43221, Suez, Egypt; 5 Department of Information Systems, Faculty of Computers and Information, Mansoura University, P.O. Box:35516, Mansoura, Egypt

**Keywords:** Hybrid deep learning, GRU–MLP model, House price prediction, Metaheuristic optimization, Binary whale optimization algorithm (BWOA), Ant colony optimization (ACO), Hyperparameter tuning, Regression modeling, Engineering, Mathematics and computing

## Abstract

Accurate house price prediction is essential for real estate valuation, investment planning, and intelligent property decision-support systems. This study proposes an optimized hybrid deep learning framework that integrates a Gated Recurrent Unit and Multilayer Perceptron model with the Binary Whale Optimization Algorithm for feature selection and Ant Colony Optimization for hyperparameter tuning. The proposed framework was evaluated using a publicly available Kaggle house price regression dataset containing 500 housing records with structural, locational, and amenity-related attributes. The dataset was divided into training, validation, and testing subsets using a 70:20:10 ratio, and leakage-free normalization was applied using only the training data. Experimental results show that the proposed BWOA–ACO–GRU–MLP model outperformed standalone GRU, MLP, CNN, LSTM, and BiLSTM models. It achieved an MSE of 0.0146, MAE of 0.1051, RMSE of 0.1208, MAPE of 0.0112, MedAE of 0.0969, and an R^2^ of 99.04%. These results demonstrate that combining feature selection, hyperparameter optimization, and hybrid neural regression improves prediction accuracy and model stability for house price estimation. The proposed framework provides a reliable data-driven approach for smart real estate valuation applications.

## Introduction

Housing price prediction is currently considered one of the most prominent topics within intelligent real estate analytics since the property evaluation is relevant to many processes, including investment, appraisal, tax assessments, urban development, and housing policies. Conventional housing price modeling techniques, including hedonic pricing and regression analysis, usually lack flexibility, rely on predefined assumptions, and are characterized by low sensitivity to nonlinear interactions between various factors such as structural characteristics, spatial effects, economic indicators, and environment-related attributes^1,2^. To address the mentioned limitations, the use of machine learning and deep learning methods was proposed in numerous academic studies, since they enable the learning of complex patterns from heterogeneous data and enhance the efficiency of automated valuation models. As an illustration, Park & Bae^3^ presented machine learning algorithms for predicting housing prices and showed that data-driven methods were beneficial for real estate applications. Moreover, several other recent automated housing price valuation models confirmed the efficiency of machine learning and deep learning models.

Studies conducted have again shown that there is more to real estate price prediction than just using classical regression methods. With automated value estimation models, the use of ensembles, deep neural networks, gradient boosting, spatial information, description texts, and even explainable artificial intelligence can be utilized. Jafary et al.^1^ developed automated valuation models for land that considered physical, geographic, socio-economic, environmental, legal, and planning information about the land. As shown by the study, it was possible for machine learning techniques like XGBoost to produce better results than classical methods. Baur et al.^4^, on the other hand, examined the use of machine learning for automated real estate valuation, considering property descriptions.

Despite this progress, predicting house prices still poses some challenges as there are many interacting variables that determine the cost of housing, such as house size, the number of rooms in a house, age of construction, quality of the location, presence of amenities, and proximity to cities. The relationships among these variables might be complex in nature and require more advanced approaches than traditional models to obtain highly accurate predictions. Recently, many studies on housing prices have revealed that machine learning algorithms can model nonlinear market dynamics and heterogeneities well, and explainable models have also been developed to understand how the location and properties influence prices^5,6^.

The other major challenge is model optimization. Machine learning and deep learning models are very dependent on hyperparameters like learning rate, the number of hidden layers, dropout ratio, batch size, and architecture depth. Hyperparameters selected wrongly will cause overfitting, instabilities in convergence, and poor generalization ability. Optimization algorithms are therefore critical in improving the accuracy of forecasts in hybrid models^7^. For example, Zhan et al.^8^ suggested a hybrid Bayesian optimization algorithm using stacking, bagging, and transformer models for house price prediction. Metaheuristic optimization has proven effective in deep learning problems by discovering the best configurations compared to human intervention.

The deep learning architecture, including GRU, LSTM, CNN, and MLP, is extensively used in predicting tasks because of its ability to model nonlinear relations and interactions between different features. GRU networks are especially helpful because they offer a simplified recurrent architecture compared to LSTM, but still have the capacity to model sequence dependencies. Meanwhile, MLP is a powerful universal function approximation tool for tabular regression tasks that learns nonlinear functions mapping input variables to continuous output values. Recent hybrid models demonstrate that using GRU in combination with other neural network models improves prediction performance in nonlinear prediction tasks. At the same time, optimizing GRU networks leads to good results in real-world prediction tasks^9,10^.

However, the existing literature still presents several limitations. First, many studies rely mainly on standalone machine learning models, such as Random Forest, XGBoost, Support Vector Regression, or standard neural networks, without sufficiently investigating hybrid recurrent–feedforward architectures for house price regression. Second, although optimization algorithms have been applied in several forecasting domains, the integration of the Binary Whale Optimization Algorithm and Ant Colony Optimization for hyperparameter tuning in house price prediction remains underexplored. Third, many previous studies focus only on predictive accuracy but provide limited comparative analysis across deep learning baselines such as GRU, MLP, CNN, LSTM, and BiLSTM^11,12^. Fourth, some studies do not sufficiently analyze convergence behavior, training–validation stability, or error-based regression indicators such as MSE, MAE, RMSE, MAPE, MedAE, and R^2^ together. These gaps motivate the development of a hybrid optimized deep learning model that can provide more accurate, stable, and generalizable house price predictions.

This study addresses the weaknesses by proposing a GRU–MLP hybrid architecture trained using the Binary Whale Optimization Algorithm for feature selection and Ant Colony Optimization to predict house prices. Although the housing dataset used in this study is tabular and does not contain natural temporal sequences, the GRU component is employed as a gated nonlinear feature-interaction encoder rather than as a conventional time-series forecasting model. After BWOA-based feature selection, each housing record is represented as a fixed ordered vector of normalized attributes, including structural, amenity-related, and locational variables. The GRU gates allow the model to selectively retain, suppress, and combine information across these attributes, thereby learning compact feature representations before the final regression stage. The MLP component is then used to map the learned representation to the continuous house price output. To assess the model’s efficacy in predicting house prices, an open-source housing price regression dataset from Kaggle with 500 instances is used. This data set incorporates numerous attributes related to structural and location characteristics of houses, such as square feet, number of bedrooms, number of bathrooms, number of floors, year built, presence of a garden, pool, garage size, location rating, proximity to downtown, and price. The data set is partitioned into training, validation, and test sets in the ratio of 70:20:10. The findings obtained reveal that the proposed GRU–MLP model performs better than other models, achieving MSE = 0.0146, MAPE = 0.0112, MedAE = 0.0969, MAE = 0.1051, RMSE = 0.1208, and R^2^ = 99.04%.

The main contributions of this study are summarized as follows:

1. Proposing a hybrid deep learning model combining GRU and MLP for enhanced nonlinear regression in house price prediction.
2. Integrating BWOA for feature selection, effectively reducing redundancy and improving predictive performance.
3. Applying ACO for hyperparameter tuning to enhance convergence and model generalization.
4. Conducting a comprehensive comparative analysis with multiple deep learning baselines.
5. Demonstrating the effectiveness of combining feature selection and optimization techniques in a unified predictive framework.

The remainder of this paper is organized as follows. "Related works" section reviews related work on house price prediction and optimization techniques. "Materials and methods" section presents the proposed methodology, including BWOA-based feature selection and ACO-based hyperparameter tuning. "Experimental setup" section describes the experimental setup and evaluation metrics. "Results and discussion" section discusses the results and comparative analysis. Finally, "Conclusion and future work" section concludes the paper and outlines future research directions.

## Related works

There have been considerable recent advances in house price prediction owing to the ever-increasing sophistication of the real estate market and the availability of data. Among traditional approaches for predicting real estate prices are hedonic regression models and index methods that have been popular over the years. However, their major limitation is that they are not efficient at modeling the complex relationship between various factors affecting house prices. Hence, there has been an exploration of numerous methods based on machine learning and deep learning algorithms to improve predictive performance and model robustness. This has also led to new research areas, including feature engineering and model optimization, in order to boost performance even further and increase interpretability. There still remain some problems with high-dimensional data, selecting appropriate features, avoiding overfitting, and defining model objectives consistent with real-world application scenarios. In this regard, this paper will review the most related studies on house price prediction utilizing machine learning models, hybrid modeling techniques, feature selection approaches, and optimization-based methods.

The research conducted by Adetunji et al.^13^. It is about the prediction of house prices while considering the drawbacks of conventional measures like the House Price Index (HPI). According to the research, the prediction of house prices should not rely solely on the identification of overall market trends, but it should also consider predicting the prices and/or the price ranges of properties. Predicting a variance or range would make sense and be applicable in practical situations, which made the problem a matter of classification. In order to mitigate the shortcomings of HPI, the researchers considered the inclusion of more attributes about the property and location of each property. The Random Forest algorithm was implemented using the Boston Housing data set containing 506 instances of 14 attributes available at the UCI Repository. The results have shown that the implemented method performed reliably by providing predicted prices almost equal to actual prices in error margins of ± 5%.

The problem of the complexity of property evaluation was discussed by Zaki et al.^14^. In this case, the complexity is caused by the impact of space, time, and economy, making it difficult to estimate the price accurately. Although the classical methods for evaluating property, including the hedonic regression model, have been applied frequently, they still face the drawbacks of limited accuracy and failure to address non-linear problems. Thus, the paper proposed a novel machine learning approach incorporating the Extreme Gradient Boosting algorithm with the outlier sum-statistic method. Both XGBoost and hedonic regression models were constructed based on 13 independent variables to predict housing prices. The experiment revealed that the XGBoost model exhibited more accurate prediction (84.1%) than the classic regression model (42%).

Sharma et al.^15^ investigated house price prediction as a regression problem, emphasizing that property valuation is influenced not only by structural attributes but also by neighborhood and environmental factors. The study utilized the Ames Housing dataset to evaluate multiple machine learning models, including XGBoost, Support Vector Regressor, Random Forest, Multilayer Perceptron, and Multiple Linear Regression. In addition to predictive modeling, the research focused on identifying the most influential factors affecting housing prices, providing interpretability alongside performance. Experimental results revealed that XGBoost achieved the highest predictive accuracy among the evaluated models, demonstrating its superiority in capturing complex nonlinear relationships in housing data.

Mora-García et al.^16^ explored the application of machine learning techniques for house price prediction using large-scale georeferenced microdata, with a particular focus on analyzing the impact of the COVID-19 pandemic on real estate markets. The study implemented a comprehensive framework that included data preprocessing, feature engineering, hyperparameter optimization, model evaluation, and interpretability analysis. Multiple machine learning models were evaluated, including boosting-based methods (Gradient Boosting Regressor, XGBoost, and LightGBM) and bagging-based approaches (Random Forest and Extra Trees), alongside a traditional linear regression baseline. The experimental analysis, conducted on real estate data from Alicante, Spain, demonstrated that machine learning models significantly outperform linear models due to their ability to capture complex nonlinear relationships in housing data. Furthermore, boosting algorithms exhibited superior performance and reduced overfitting compared to bagging methods.

Zhan et al.^8^ proposed an advanced hybrid framework for house price prediction aimed at improving forecasting accuracy and model stability in complex real estate markets. The study introduced novel hybrid models that integrate Bayesian Optimization with ensemble learning techniques, including stacking, bagging, and Transformer-based architectures. These models leverage Bayesian Optimization for efficient hyperparameter tuning, enabling enhanced predictive performance across diverse scenarios. A key contribution of the study is the construction of a large-scale, multi-source dataset comprising approximately 1.9 million real estate transactions from the Hong Kong market spanning over two decades, providing a robust benchmark for model evaluation. The proposed models were rigorously compared against 18 baseline methods using 13 evaluation metrics, along with statistical significance tests such as Friedman, Iman–Davenport, and Nemenyi post-hoc analysis. Experimental results demonstrated that the hybrid stacking-based model (HBOS-CatBoost) achieved superior performance, with notable reductions in RMSE compared to other hybrid configurations.

Akyüz et al.^17^ proposed a dynamic hybrid framework for house price prediction designed to address the heteroscedastic and highly variable nature of real estate data. The study integrates multiple techniques, including linear regression, clustering analysis, nearest neighbor classification, and Support Vector Regression (SVR), within a unified pipeline where the output of one stage serves as the input to subsequent stages. The approach first partitions housing data into homogeneous clusters, then classifies new instances into appropriate groups, and finally applies specialized regression models for each cluster to improve prediction accuracy. The proposed model was evaluated using real-world housing data collected from the Kadıköy district in Istanbul, as well as a benchmark Kaggle dataset with diverse property features. Comparative analysis against several baseline models, including linear regression, Lasso, Ridge, SVR, AdaBoost, decision tree, random forest, and XGBoost, demonstrated that the hybrid approach achieved superior performance in terms of RMSE, MAPE, and adjusted R^2^.

Hjort et al.^18^ investigated the performance evaluation of Automated Valuation Models (AVMs) in the context of real estate price estimation, emphasizing the discrepancy between traditional optimization objectives and industry-relevant evaluation metrics. While most machine learning models, including gradient boosted trees and random forests, are typically trained to minimize mean squared error, the study argued that real-world financial institutions assess model performance based on the proportion of predictions falling within predefined error margins (± 10% and ± 20%). To address this gap, the authors proposed a modified loss function aligned with these practical evaluation criteria and applied it within a gradient boosted tree framework. Using a large dataset comprising 126,719 housing transactions from the Norwegian market, the results demonstrated improved predictive performance, increasing the proportion of predictions within ± 20% error from 89.4 to 90.0%. Furthermore, combining models optimized with different loss functions yielded additional improvements, achieving 90.4% accuracy within the same range.

Lahmiri et al.^19^ investigated the effectiveness of advanced artificial intelligence and machine learning techniques for house price prediction in response to increasing demand for accurate valuation systems following global financial and real estate crises. The study compared multiple predictive approaches, including boosting ensemble regression trees, support vector regression, and Gaussian process regression, within a rigorous framework incorporating Bayesian optimization and ten-fold cross-validation for optimal parameter tuning. Using multiple evaluation metrics, the results demonstrated that boosting ensemble regression trees achieved the highest predictive performance, followed by Gaussian process regression and support vector regression. Furthermore, all proposed models significantly outperformed traditional approaches such as artificial neural networks and multivariate regression.

Sibindi et al.^20^ examined the complexity of property valuation, emphasizing the challenges associated with modeling housing prices due to spatial, temporal, and economic dependencies. To improve predictive accuracy, the study explored the integration of machine learning techniques with traditional valuation approaches. Specifically, the authors combined the Extreme Gradient Boosting (XGBoost) algorithm with an outlier sum-statistic (OS) method to enhance model robustness and handle irregularities in housing data. The proposed framework was evaluated using 13 input variables and compared against the conventional hedonic pricing model. Experimental results demonstrated that the XGBoost-based approach significantly outperformed the traditional method, achieving an accuracy of 84.1% compared to 42% for hedonic regression.

Rey-Blanco et al.^21^ addressed the critical role of location factors in house price prediction by proposing an advanced methodology for constructing comprehensive accessibility-based location indices. The study highlighted the limitations of traditional location modeling, including the lack of consensus on index construction, difficulty in selecting relevant variables, and the need for granular and up-to-date datasets. To overcome these challenges, the authors developed a data-driven framework that generates car and walk accessibility indices using automated search techniques to identify optimal variable combinations. Principal Component Analysis (PCA) was subsequently applied to ensure orthogonality and reduce multicollinearity among the generated indices. The proposed indices were evaluated using both regression models and random forest algorithms on a large real estate dataset from Madrid. Experimental results demonstrated significant improvements in predictive accuracy, with gains of 13% for regression models and 21.6% for random forests, alongside a notable reduction in spatial autocorrelation by approximately 35%.

Alzain et al.^22^ investigated the application of artificial neural networks (ANN) for house price prediction, emphasizing the importance of accurate valuation models in supporting economic planning, investment decisions, and government regulation. The study developed an ANN-based predictive framework tailored to the Saudi Arabian real estate market, utilizing housing data collected from major cities, including Riyadh, Jeddah, Dammam, and Al-Khobar. The proposed model was designed to capture complex nonlinear relationships between housing attributes and price distributions across different geographic regions. Experimental results demonstrated that the ANN model achieved high predictive accuracy, with predicted values closely matching actual market prices. Comparative analysis with existing prediction methods further confirmed the effectiveness of the proposed approach.

The reviewed studies collectively demonstrate that machine learning and hybrid models have significantly improved house price prediction by capturing nonlinear relationships, leveraging ensemble learning (e.g., XGBoost, boosting trees), incorporating spatial and accessibility features, and applying optimization techniques such as Bayesian tuning. However, most existing works focus either on model development (e.g., ANN, boosting, hybrid clustering–regression frameworks) or optimization strategies independently, with limited integration of feature selection and hyperparameter optimization within a unified pipeline. In addition, several studies rely on traditional evaluation settings or lack a comprehensive comparative analysis across deep learning architectures. In contrast, the present study advances the field by proposing a fully integrated framework that combines Binary Whale Optimization Algorithm (BWOA) for feature selection, Ant Colony Optimization (ACO) for hyperparameter tuning, and a hybrid GRU–MLP deep learning model. This unified approach addresses key limitations in prior work by simultaneously reducing feature redundancy, improving model convergence, and enhancing predictive accuracy, thereby providing a more robust, efficient, and scalable solution for house price prediction.

## Materials and methods

### Dataset description

This study uses a publicly available house price regression dataset obtained from Kaggle^23^. The dataset is designed for supervised regression analysis, where the objective is to predict the continuous target variable, Price, based on several structural, locational, and amenity-related housing attributes. The dataset contains 500 housing records and 11 variables, including 10 independent input features and one dependent output variable. According to the dataset summary, the features include property size, number of rooms, construction year, availability of facilities such as garden and pool, garage size, location quality, and distance from the city center. The dataset was divided into training, validation, and testing subsets using a 70:20:10 ratio, allowing the model to learn from the training data, optimize performance using the validation set, and finally evaluate generalization on an unseen test set. This partitioning strategy supports a fair assessment of the proposed GRU–MLP model and the baseline models, while reducing the risk of overfitting to the training data.

Although the dataset provides useful structural, locational, and amenity-related housing attributes, its size is relatively limited, with only 500 records. Therefore, the results obtained from a single holdout test set should not be interpreted as definitive evidence of broad market-level generalization. To reduce the dependence of the evaluation on one random split, the experimental protocol was extended using repeated k-fold cross-validation. This strategy allows all samples to contribute to testing across different folds and provides a more reliable estimate of model stability than a single 50-sample test subset.

Table 1 presents the descriptive statistical summary of the housing dataset used in this study, including 500 records for each variable. The dataset contains structural features such as Square_Feet, Num_Bedrooms, Num_Bathrooms, Num_Floors, and Year_Built, as well as amenity and location-related variables such as Has_Garden, Has_Pool, Garage_Size, Location_Score, and Distance_to_Center. The average house size is approximately 174.64 square feet, while the mean number of bedrooms and bathrooms is 2.96 and 1.98, respectively. The target variable, Price, shows considerable variation, ranging from 276,892.47 to 960,678.27, with a mean of 582,209.63 and a standard deviation of 122,273.39. This wide price range indicates that the dataset contains diverse housing samples, making it suitable for evaluating regression models. Moreover, the variation in location score, distance to the city center, garage size, and amenity availability suggests that both structural and spatial factors may contribute significantly to house price prediction, supporting the need for an optimized feature selection and deep learning-based prediction framework.

**Table 1:** Descriptive statistical summary of the housing price prediction dataset.

Variable	Count	Mean	Std. Dev	Min	Median	Max
Square_Feet	500	174.64	74.67	51.27	178.29	298.24
Num_Bedrooms	500	2.96	1.44	1.00	3.00	5.00
Num_Bathrooms	500	1.98	0.82	1.00	2.00	3.00
Num_Floors	500	1.96	0.80	1.00	2.00	3.00
Year_Built	500	1957.60	35.49	1900.00	1959.00	2022.00
Has_Garden	500	0.54	0.50	0.00	1.00	1.00
Has_Pool	500	0.49	0.50	0.00	0.00	1.00
Garage_Size	500	30.17	11.58	10.00	30.00	49.00
Location_Score	500	5.16	2.85	0.00	5.21	10.00
Distance_to_Center	500	10.47	5.59	0.06	10.89	19.93
Price	500	582209.63	122273.39	276892.47	574724.11	960678.27

**Fig. 1:**
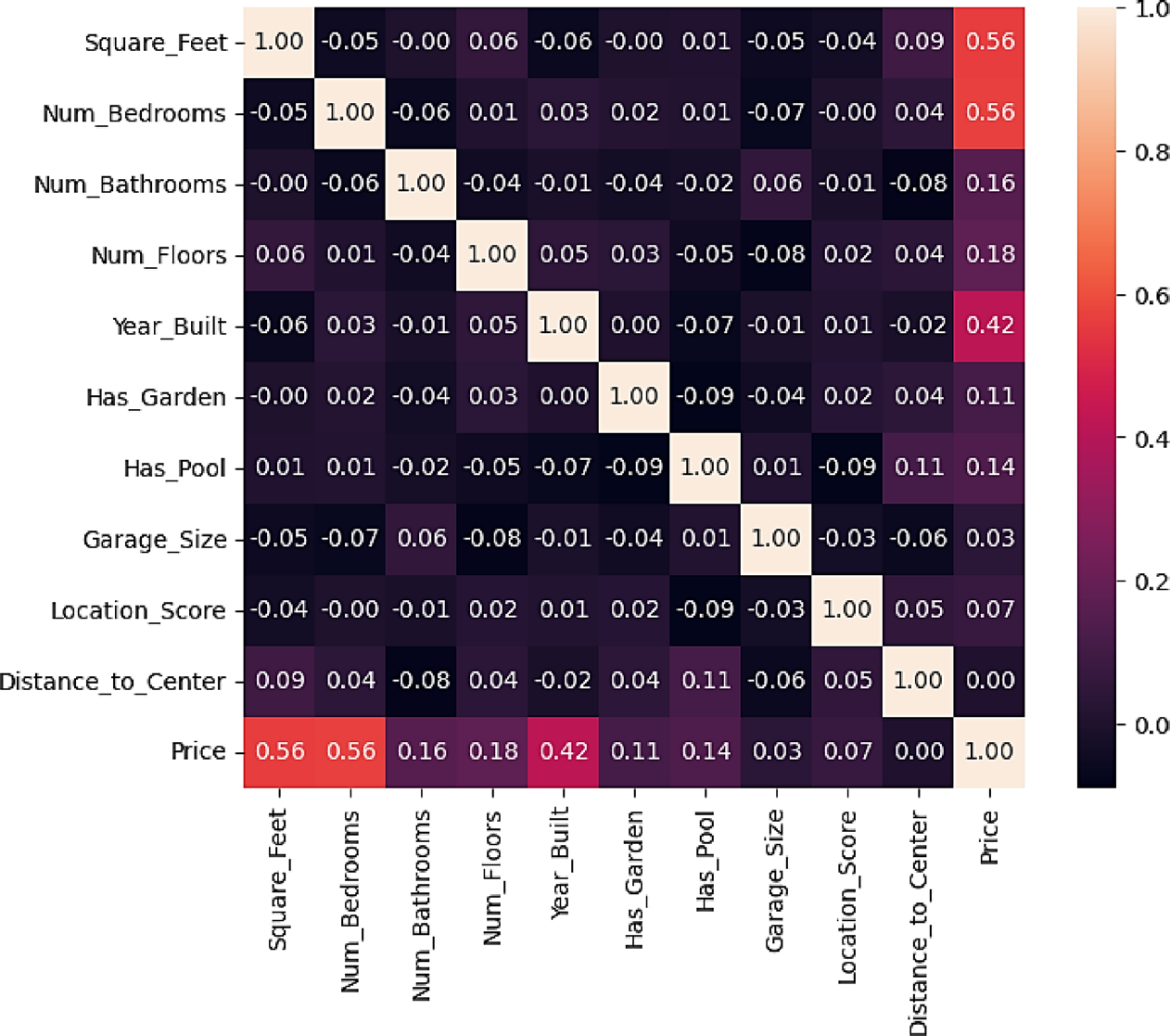
Correlation heatmap showing relationships among housing attributes and house price.

Figure 1 illustrates the correlation matrix among the input variables and the target variable Price. The results show that Square_Feet and Num_Bedrooms have the strongest positive correlations with house price, each reaching approximately 0.56, indicating that larger houses and houses with more bedrooms tend to have higher prices. Year_Built also shows a moderate positive relationship with price (0.42), suggesting that newer properties may be associated with higher market value. Other variables, such as Num_Floors (0.18), Num_Bathrooms (0.16), Has_Pool (0.14), and Has_Garden (0.11), show weaker but still positive associations with price. In contrast, Garage_Size, Location_Score, and Distance_to_Center exhibit very weak correlations with the target variable in this dataset.

**Fig. 2:**
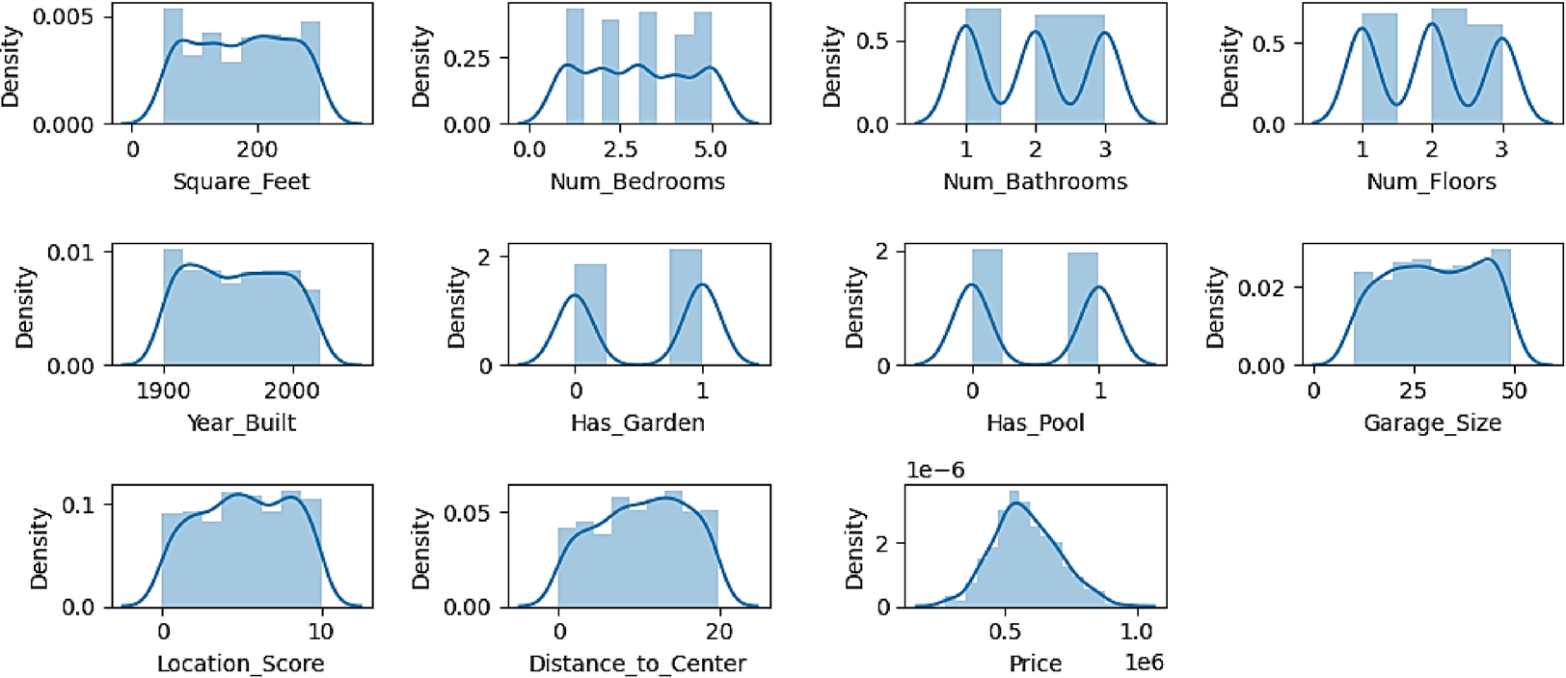
Density distribution plots of structural, locational, amenity-related, and target variables in the housing dataset.

Figure 2 presents the density distributions of the housing dataset variables, providing an overview of how each feature is distributed before model training. The plots show that several structural variables, such as Num_Bedrooms, Num_Bathrooms, and Num_Floors, follow discrete multi-peak distributions because they represent count-based property attributes. The binary amenity variables Has_Garden and Has_Pool show two distinct peaks at 0 and 1, reflecting the absence or presence of these facilities. Continuous variables such as Square_Feet, Garage_Size, Location_Score, and Distance_to_Center display broader distributions, indicating variability among housing samples. The target variable Price appears approximately bell-shaped with a concentration around the middle price range, suggesting that most properties fall within a moderate price interval while fewer samples exist at the extreme low and high price ranges.

**Fig. 3:**
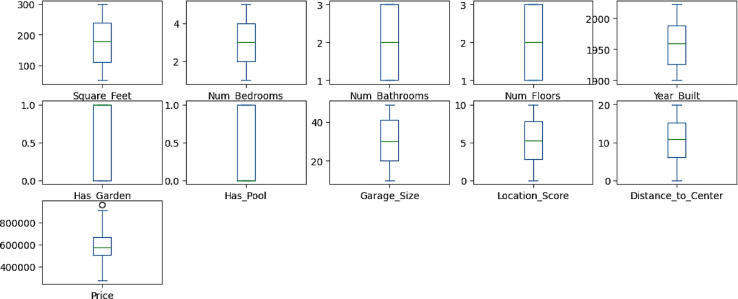
Boxplot analysis of housing dataset features and target price distribution.

Figure 3 presents the boxplot analysis of the housing dataset variables, showing the median, interquartile range, spread, and potential outliers for each feature. The plots indicate that Square_Feet, Garage_Size, Location_Score, and Distance_to_Center have relatively wide distributions, reflecting variability in property size, garage capacity, location quality, and accessibility. Count-based variables such as Num_Bedrooms, Num_Bathrooms, and Num_Floors show limited discrete ranges, while Has_Garden and Has_Pool confirm their binary nature through values concentrated at 0 and 1. The Year_Built variable spans from older to newer properties, indicating temporal diversity in the dataset. For the target variable Price, the boxplot shows a central concentration around the median price, with a few high-value outliers above the upper whisker, suggesting the presence of some expensive properties.

**Fig. 4:**
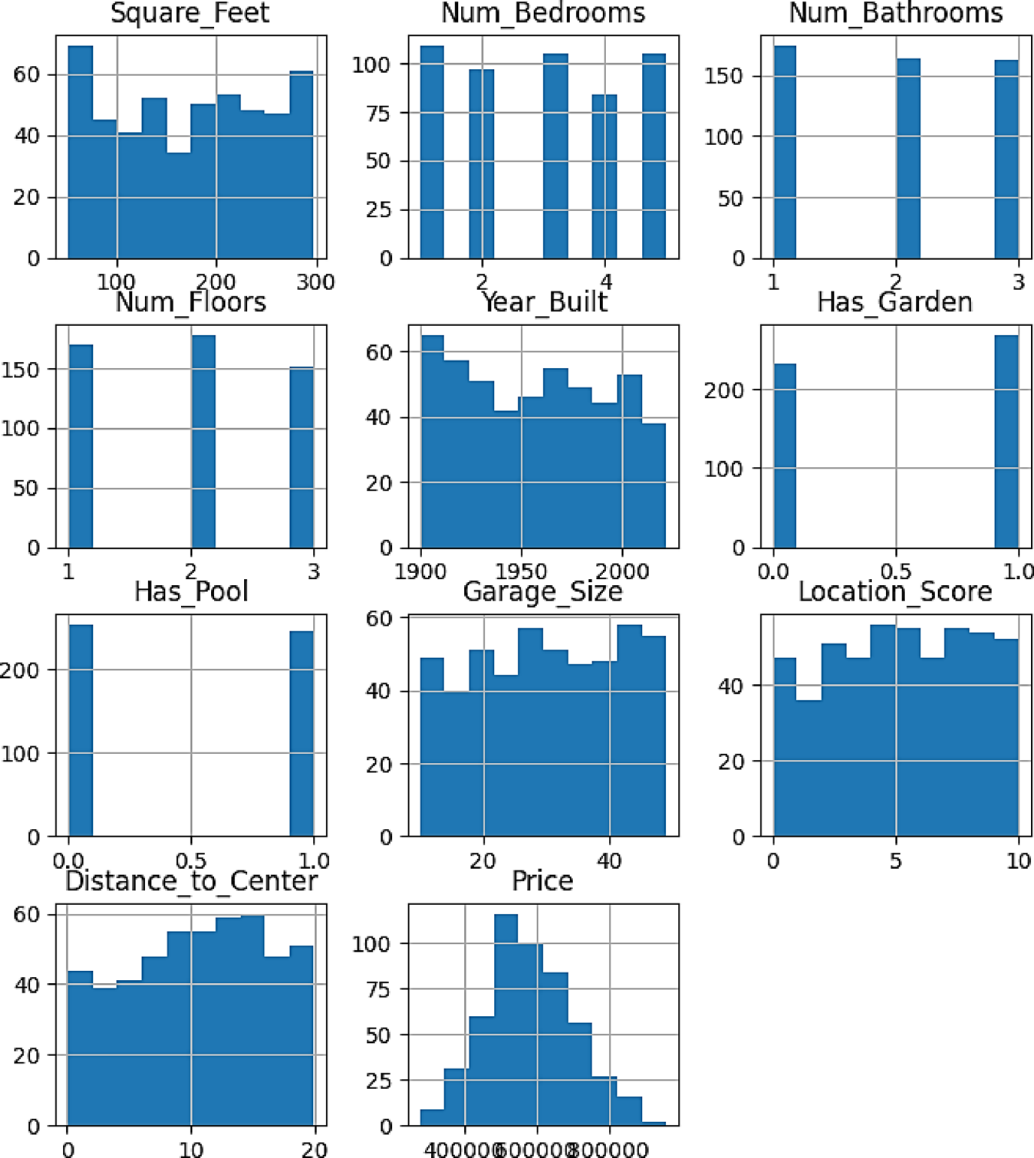
Histogram distributions of housing dataset features and target house price.

Figure 4 shows the histogram-based frequency distribution of all housing attributes and the target variable Price, providing a clearer view of how the dataset samples are distributed across different value ranges. The structural variables show diverse patterns: Square_Feet is spread across a wide interval, indicating variation in house sizes, while Num_Bedrooms, Num_Bathrooms, and Num_Floors appear as discrete distributions because they represent count-based features. The binary variables Has_Garden and Has_Pool are divided into two categories, confirming the balanced presence and absence of these amenities in the dataset. Continuous variables such as Garage_Size, Location_Score, and Distance_to_Center show broad distributions, suggesting that the dataset includes properties with different garage capacities, location qualities, and distances from the city center. The Price histogram follows an approximately normal distribution, with most houses concentrated around the middle price range and fewer observations at the lower and upper extremes.

### Data preprocessing

Data preprocessing was conducted to prepare the housing dataset for reliable feature selection, hyperparameter optimization, and regression modeling. The dataset contains numerical, binary, structural, locational, and amenity-related variables; therefore, preprocessing was necessary to ensure data consistency, reduce scale bias, prevent data leakage, and improve the stability of the proposed BWOA–ACO–GRU–MLP framework.

#### Dataset Representation

The housing dataset can be represented as a supervised regression dataset:

1 $$D=\{({x}_{i},{y}_{i}){\}}_{i=1}^{n}$$

where ${x}_{i}\in {\mathbb{R}}^{d}$ denotes the input feature vector of the $i$-th house, ${y}_{i}\in {\mathbb{R}}$ represents the corresponding house price, $n$ is the number of samples, and $d$ is the number of input features.

Each input vector is defined as:

2 $${x}_{i}=[{x}_{i1},{x}_{i2},{x}_{i3},\dots ,{x}_{id}]$$

where the input attributes include Square_Feet, Num_Bedrooms, Num_Bathrooms, Num_Floors, Year_Built, Has_Garden, Has_Pool, Garage_Size, Location_Score, and Distance_to_Center. The target variable is Price.

The prediction problem is formulated as learning a nonlinear mapping function between housing attributes and house prices:

3 $${\widehat{y}}_{i}=f({x}_{i};\theta )$$

where ${\widehat{y}}_{i}$ is the predicted house price and $\theta$ denotes the learnable parameters of the prediction model.

#### Missing Value Inspection

Missing-value inspection was performed to ensure data completeness before model training. For each feature ${x}_{j},$ the missing-value ratio was computed as:

4 $$MR_{j} = \frac{{\mathop \sum \nolimits_{i = 1}^{n} I\left( {x_{ij} = \emptyset } \right)}}{n}$$

where $M{R}_{j}$ denotes the missing-value ratio of the $j$-th feature, $I(\cdot )$ is an indicator function, and $\emptyset$ represents a missing value.

If missing values are detected in numerical variables, they can be replaced using the median value of the corresponding feature:

5 $$x_{ij}^{*} = \left\{ {\begin{array}{*{20}c} {x_{ij} ,} & {{\text{if }}\,x_{ij} \, \ne \emptyset } \\ {{\mathrm{median}}\,\left( {x_{j} } \right),} & {{\text{if }}\,x_{ij} \, = \emptyset } \\ \end{array} } \right.$$

where ${x}_{ij}^{*}$ is the imputed value. In this study, all variables had 500 valid observations, indicating no missing values in the dataset.

#### Feature–target separation

After checking data completeness, the dataset was divided into the input feature matrix $X$ and the target vector $Y$. The feature matrix is represented as:

6 $$X=\left[\begin{array}{cccc}{x}_{11}& {x}_{12}& \cdots & {x}_{1d}\\ {x}_{21}& {x}_{22}& \cdots & {x}_{2d}\\ \vdots & \vdots & \ddots & \vdots \\ {x}_{n1}& {x}_{n2}& \cdots & {x}_{nd}\end{array}\right]$$

and the target vector is defined as:

7 $$Y=\left[\begin{array}{c}{y}_{1}\\ {y}_{2}\\ \vdots \\ {y}_{n}\end{array}\right]$$

where:

$X\in {\mathbb{R}}^{n\times d},Y\in {\mathbb{R}}^{n\times 1}$, The feature matrix $X$ was used as the input to the baseline and proposed models, while $Y$ represented the continuous house price output.

#### Data splitting

The dataset was initially divided into training, validation, and testing subsets using a 70:20:10 ratio, corresponding to 350 training samples, 100 validation samples, and 50 testing samples. However, because the test subset contains only 50 samples, relying exclusively on this split may lead to high variance in the reported performance. Therefore, repeated k-fold cross-validation was additionally used to obtain a more robust performance estimate. In each repetition, the dataset was partitioned into k folds, where k − 1 folds were used for model development, and the remaining fold was used for testing^24,25^. The training portion within each fold was further divided into training and validation subsets for BWOA-based feature selection, ACO-based hyperparameter tuning, and early convergence monitoring. All preprocessing operations, including scaling, feature selection, and hyperparameter optimization, were fitted only on the training data of each fold and then applied to the validation and test folds. This protocol prevents data leakage and provides a more reliable estimate of generalization performance on a small dataset.

The split can be mathematically expressed as:

8 $$D={D}_{train}\cup {D}_{val}\cup {D}_{test}$$

9 $$D_{{train}} \cap D_{{val}} \cap D_{{test}} = \emptyset$$

The subset sizes are defined as:

$$\mid {D}_{train}\mid =0.70n$$

$$\mid {D}_{val}\mid =0.20n$$

$$\mid {D}_{test}\mid =0.10n$$

Since the dataset contains $n=500$ samples, the resulting split is:

10 $$\mid {D}_{train}\mid =350,\mid {D}_{val}\mid =100,\mid {D}_{test}\mid =50$$

This splitting strategy ensures that the final model is evaluated on unseen data and reduces the risk of overly optimistic performance estimation.

#### Feature scaling and normalization

Because the dataset contains variables with different numerical ranges, feature scaling was applied to reduce scale bias during neural network training and optimization. For example, Price, Year_Built, and Square_Feet have larger numerical magnitudes than binary variables such as Has_Garden and Has_Pool. Therefore, min–max normalization was used to transform numerical features into a comparable scale.

For each feature ${x}_{j}$, min–max normalization is defined as:

11 $${x}_{ij}^{norm}=\frac{{x}_{ij}-{x}_{j}^{min}}{{x}_{j}^{max}-{x}_{j}^{min}}$$

where ${x}_{ij}^{norm}$ is the normalized value of the $j$-th feature for sample $i$, ${x}_{j}^{min}$ is the minimum value of the feature, and ${x}_{j}^{max}$ is the maximum value of the feature.

After normalization, all values are scaled into the range:

12 $${x}_{ij}^{norm}\in [\mathrm{0,1}]$$

#### Target variable normalization

The target variable Price was also normalized because it has a large numerical range. Normalizing the target variable helps stabilize model training, reduce gradient instability, and improve convergence speed.

The normalized target value is computed as:

13 $${y}_{i}^{norm}=\frac{{y}_{i}-{y}^{min}}{{y}^{max}-{y}^{min}}$$

where ${y}_{i}^{norm}$ is the normalized price value, and ${y}^{min}$ and ${y}^{max}$ are the minimum and maximum price values.

After prediction, the normalized output can be transformed back into the original price scale using inverse normalization:

14 $${\widehat{y}}_{i}={\widehat{y}}_{i}^{norm}({y}^{max}-{y}^{min})+{y}^{min}$$

where ${\widehat{y}}_{i}^{norm}$ is the normalized predicted value and ${\widehat{y}}_{i}$ is the predicted house price in the original scale.

The target variable Price was normalized using min–max scaling before model training. Therefore, the regression loss and the reported error-based performance metrics were calculated on the normalized target scale. This choice was adopted to ensure numerical stability, reduce the effect of the large price range, and allow direct comparison among models trained under the same preprocessing conditions. Accordingly, MSE, RMSE, MAE, MAPE, and MedAE reported in the experimental results are normalized-scale metrics.

#### Leakage-free scaling strategy

To avoid data leakage, normalization parameters were computed using only the training subset. The same scaling parameters were then applied to the validation and testing subsets. This step prevents information from the validation or testing data from influencing the preprocessing process.

For each feature in the training set, the minimum and maximum values are computed as:

15 $${x}_{j,train}^{min}=\mathrm{m}\mathrm{i}\mathrm{n}({x}_{j}^{train})$$

16 $${x}_{j,train}^{max}=\mathrm{m}\mathrm{a}\mathrm{x}({x}_{j}^{train})$$

Then, any sample from the validation or testing subset is scaled as:

17 $${x}_{ij}^{scaled}=\frac{{x}_{ij}-{x}_{j,train}^{min}}{{x}_{j,train}^{max}-{x}_{j,train}^{min}}$$

This leakage-free preprocessing strategy ensures that model evaluation reflects realistic performance on unseen data.

#### BWOA-based feature selection

Feature selection was performed using the BWOA^26,27^ to identify the most relevant housing attributes and remove redundant or weakly informative features. In BWOA, each candidate solution is represented as a binary vector:

18 $${S}_{k}=[{s}_{k1},{s}_{k2},\dots ,{s}_{kd}]$$

where ${S}_{k}$ denotes the $k$-th whale candidate solution, and:

19 $${s}_{kj}\in \{\mathrm{0,1}\}$$

A value of ${s}_{kj}=1$ indicates that the $j$-th feature is selected, while ${s}_{kj}=0$ indicates that the feature is excluded.

The selected feature subset is represented as:

20 $${X}_{{S}_{k}}=\{{x}_{j}\mid {s}_{kj}=1,\hspace{0.25em} j=\mathrm{1,2},\dots ,d\}$$

The total number of selected features is computed as:

21 $$\mid {S}_{k}\mid =\sum_{j=1}^{d}{s}_{kj}$$

The fitness function was designed to balance prediction error and feature reduction:

22 $$Fitness({S}_{k})=\alpha \times MSE({S}_{k})+(1-\alpha )\times \frac{\mid {S}_{k}\mid }{d}$$

where $MSE({S}_{k})$ is the validation mean-squared error obtained using the selected feature subset, $\frac{\mid {S}_{k}\mid }{d}$ is the ratio of selected features to the total number of features, and $\alpha \in [\mathrm{0,1}]$ controls the trade-off between prediction accuracy and feature reduction.

The validation mean squared error is calculated as:

23 $$MSE=\frac{1}{m}\sum_{i=1}^{m}({y}_{i}-{\widehat{y}}_{i}{)}^{2}$$

where $m$ is the number of validation samples, ${y}_{i}$ is the actual normalized price, and ${\widehat{y}}_{i}$ is the predicted normalized price.

Because the original Whale Optimization Algorithm produces continuous position values, a transfer function was used to convert continuous positions into binary feature-selection decisions. The sigmoid transfer function is defined as:

24 $$T(z)=\frac{1}{1+{e}^{-z}}$$

The binary update rule is then expressed as:

25 $${s}_{kj}^{t+1}=\left\{\begin{array}{cc}1,& if\; r\;and<T({z}_{kj}^{t+1})\\ 0,& otherwise\end{array}\right.$$

where $rand\in [\mathrm{0,1}]$ is a random value, ${z}_{kj}^{t+1}$ is the updated continuous position, and ${s}_{kj}^{t+1}$ is the binary decision for the $j$-th feature at iteration $t+1$.

All ten input variables were included in the initial BWOA search space, including Square_Feet, Num_Bedrooms, Num_Bathrooms, Num_Floors, Year_Built, Has_Garden, Has_Pool, Garage_Size, Location_Score, and Distance_to_Center. Therefore, no feature was manually excluded before optimization. The BWOA algorithm evaluated candidate feature subsets using validation MSE together with a feature-reduction penalty. Unlike simple correlation-based filtering, this wrapper-based selection procedure considers the joint contribution of variables to prediction performance. Consequently, a feature may be selected not only because it has a high individual correlation with Price, but also because it improves validation performance when combined with other variables.

#### ACO-based hyperparameter tuning

After selecting the optimal feature subset using BWOA, ACO^28^ was applied to tune the hyperparameters of the proposed GRU–MLP model. ACO searches for the best model configuration by simulating the pheromone-guided path selection behavior of ants.

Each candidate hyperparameter configuration is represented as:

26 $${H}_{q}=[{h}_{q1},{h}_{q2},\dots ,{h}_{qp}]$$

where ${H}_{q}$ denotes the $q$-th candidate configuration, and $p$ is the number of optimized hyperparameters, such as learning rate, GRU units, MLP neurons, dropout rate, batch size, and number of epochs.

The probability of selecting a hyperparameter option is calculated as:

27 $${P}_{ij}=\frac{{\left({\tau}_{ij}{)}^{\alpha }({\eta}_{ij}\right)}^{\beta }}{\sum_{l\in {A}_{i}}({\tau}_{il}{)}^{\alpha }({\eta}_{il}{)}^{\beta }}$$

where ${P}_{ij}$ is the probability of selecting option $j$ for hyperparameter $i$, ${\tau}_{ij}$ is the pheromone intensity, ${\eta}_{ij}$ is the heuristic desirability, ${A}_{i}$ is the set of available options, and $\alpha$ and $\beta$ control the influence of pheromone and heuristic information.

After evaluating candidate configurations, pheromone values are updated as:

28 $${\tau}_{ij}^{t+1}=(1-\rho ){\tau}_{ij}^{t}+\Delta {\tau}_{ij}$$

where $\rho \in (\mathrm{0,1})$ is the pheromone evaporation rate and $\Delta {\tau}_{ij}$ represents the pheromone deposited according to the quality of the solution.

The pheromone deposit is defined as:

29 $$\Delta {\tau}_{ij}=\frac{Q}{MSE({H}_{q})}$$

where $Q$ is a positive constant and $MSE({H}_{q})$ is the validation error obtained by the candidate hyperparameter configuration ${H}_{q}$. Lower validation error produces higher pheromone reinforcement, increasing the probability of selecting effective hyperparameter values in later iterations.

#### Final training data preparation

After preprocessing, feature scaling, BWOA-based feature selection, and ACO-based hyperparameter tuning, the optimized dataset was used to train the proposed GRU–MLP model. The training objective was to minimize the regression error between the actual and predicted normalized prices.

The loss function is defined as:

30 $$\mathcal{L}(\theta )=\frac{1}{{n}_{train}}\sum_{i=1}^{{n}_{train}}({y}_{i}^{norm}-{\widehat{y}}_{i}^{norm}{)}^{2}$$

where $\mathcal{L}(\theta )$ is the training loss, ${n}_{train}$ is the number of training samples, ${y}_{i}^{norm}$ is the normalized actual price, and ${\widehat{y}}_{i}^{norm}$ is the normalized predicted price.

Thus, the complete preprocessing pipeline ensured that the dataset was clean, properly scaled, leakage-free, dimensionally optimized, and suitable for robust house price prediction using the proposed BWOA–ACO–GRU–MLP framework.

### The proposed methodology

**Fig. 5:**
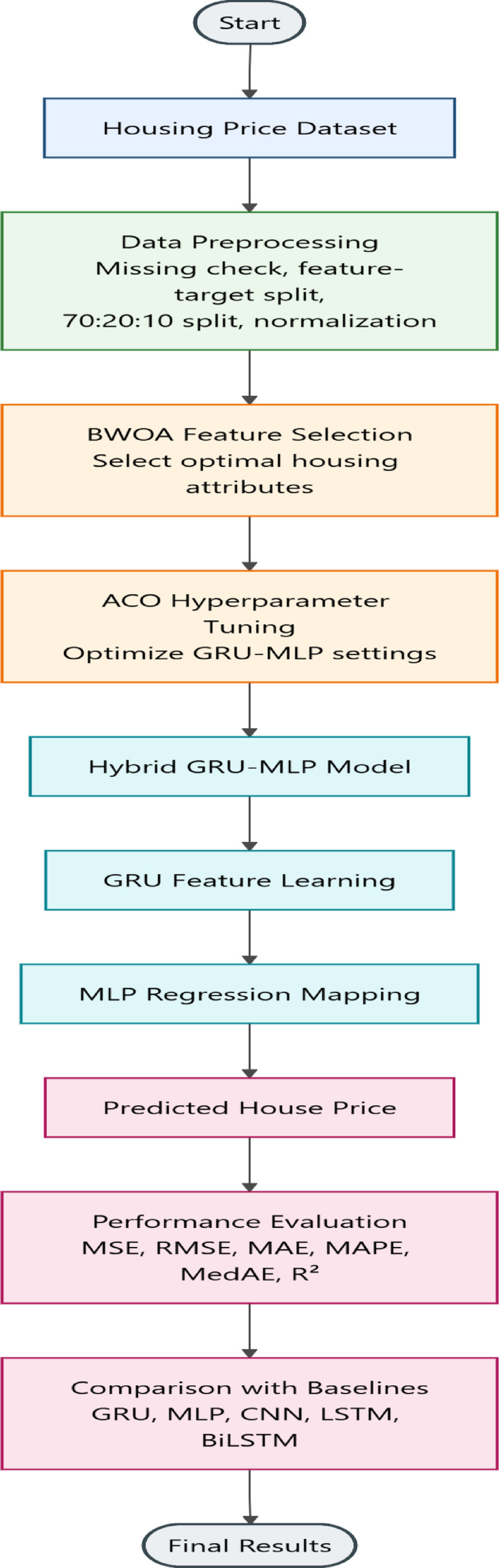
Proposed BWOA–ACO–GRU–MLP framework for optimized house price prediction.

Figure 5 illustrates the overall workflow of the proposed BWOA–ACO–GRU–MLP framework for house price prediction. As shown in Fig. 5, the process begins with the housing price dataset, followed by data preprocessing steps, including missing-value checking, feature–target separation, train–validation–test splitting, and normalization. After preprocessing, the Binary WOA is applied to select the most informative housing attributes and reduce redundant features. Then, ACO is employed to optimize the hyperparameters of the hybrid GRU–MLP model. The optimized features and parameters are passed to the GRU component for nonlinear feature learning, followed by the MLP component for regression mapping and final house price prediction. Finally, the model performance is evaluated using MSE, RMSE, MAE, MAPE, MedAE, and ${R}^{2}$, and compared with baseline models including GRU, MLP, CNN, LSTM, and BiLSTM. This figure clearly summarizes the integration of feature selection, hyperparameter tuning, and hybrid deep learning into a unified predictive framework.

The proposed predictive model combines a GRU with an MLP. The GRU component is used to learn compact nonlinear feature representations, while the MLP component performs high-level regression mapping from learned representations to the final house price. The proposed predictive model combines a GRU with an MLP. Since the dataset is tabular rather than temporal, the GRU is not used to capture chronological dependencies. Instead, it is used as a gated feature-interaction encoder. After BWOA selects the most relevant variables, each sample is represented as a fixed, ordered feature vector. This vector is reshaped into a short feature sequence only for computational compatibility with the GRU layer, where each step corresponds to one selected attribute rather than one time point. The feature order is kept identical for all samples to ensure consistent representation learning. Through its update and reset gates, the GRU can adaptively control how much information from previous attributes is retained when processing the next attribute, enabling the model to learn nonlinear interactions among selected housing characteristics. The resulting hidden representation is then passed to the MLP block for final regression mapping. Let the selected input vector at sample $i$ be:

31 $${x}_{i}^{*}=[{x}_{i1}^{*},{x}_{i2}^{*},\dots ,{x}_{ir}^{*}]$$

where $r$ is the number of selected features after BWOA.

The GRU layer computes hidden representations using reset and update gates. At time step $t$, the update gate is computed as:

32 $${z}_{t}=\sigma ({W}_{z}{x}_{t}+{U}_{z}{h}_{t-1}+{b}_{z})$$

The reset gate is calculated as:

33 $${r}_{t}=\sigma ({W}_{r}{x}_{t}+{U}_{r}{h}_{t-1}+{b}_{r})$$

The candidate hidden state is defined as:

34 $${\widetilde{h}}_{t}=\mathrm{t}\mathrm{a}\mathrm{n}\mathrm{h}({W}_{h}{x}_{t}+{U}_{h}({r}_{t}\odot {h}_{t-1})+{b}_{h})$$

The final hidden state is updated as:

35 $${h}_{t}=(1-{z}_{t})\odot {h}_{t-1}+{z}_{t}\odot {\widetilde{h}}_{t}$$

where ${x}_{t}$ is the input at time step $t$, ${h}_{t-1}$ is the previous hidden state, ${z}_{t}$ is the update gate, ${r}_{t}$ is the reset gate, ${\widetilde{h}}_{t}$ is the candidate hidden state, $W$ and $U$ are weight matrices, $b$ represents bias terms, $\sigma (\cdot )$ is the sigmoid activation function, $\mathrm{t}\mathrm{a}\mathrm{n}\mathrm{h}(\cdot )$ is the hyperbolic tangent activation function, and $\odot$ denotes element-wise multiplication.

The final GRU hidden representation is passed to the MLP block:

36 $${a}^{\left(1\right)}=\phi ({W}^{\left(1\right)}{h}_{T}+{b}^{\left(1\right)})$$

37 $$a^{\left( l \right)} = \phi \left( {W^{\left( l \right)} a^{{\left( {l1} \right)}} + b^{\left( l \right)} } \right),l = 2,3, \ldots ,L$$

where ${h}_{T}$ is the final hidden state from the GRU layer, ${a}^{\left(l\right)}$ is the activation of the $l$-th MLP layer, ${W}^{\left(l\right)}$ and ${b}^{\left(l\right)}$ are the weight matrix and bias vector of layer $l$, and $\phi (\cdot )$ is the nonlinear activation function.

The final prediction layer is formulated as:

38 $${\widehat{y}}_{i}={W}_{o}{a}^{\left(L\right)}+{b}_{o}$$

where ${\widehat{y}}_{i}$ is the predicted normalized house price, ${W}_{o}$ is the output weight matrix, and ${b}_{o}$ is the output bias.

The proposed GRU–MLP model is trained by minimizing the mean squared error between actual and predicted house prices. The loss function is defined as:

39 $$\mathcal{L}(\theta )=\frac{1}{{n}_{train}}\sum_{i=1}^{{n}_{train}}({y}_{i}-{\widehat{y}}_{i}{)}^{2}$$

where ${n}_{train}$ is the number of training samples, ${y}_{i}$ is the actual normalized house price, ${\widehat{y}}_{i}$ is the predicted normalized house price, and $\theta$ denotes all trainable parameters of the GRU–MLP model.

To improve generalization and reduce overfitting, dropout can be applied in the MLP layers as:

40 $${\widetilde{a}}^{\left(l\right)}={m}^{\left(l\right)}\odot {a}^{\left(l\right)}$$

where ${m}^{\left(l\right)}$ is a binary dropout mask sampled from a Bernoulli distribution:

41 $${m}^{\left(l\right)}\sim Bernoulli(p)$$

where $p$ is the probability of retaining neurons.

The optimal model parameters are obtained as:

42 $${\theta }^{*}=\mathrm{a}\mathrm{r}\mathrm{g}\underset{\theta }{\mathrm{m}\mathrm{i}\mathrm{n}}\mathcal{L}(\theta )$$

Algorithm 1 summarizes the complete workflow of the proposed BWOA–ACO–GRU–MLP framework for house price prediction. As shown in Algorithm 1, the process begins with preprocessing the housing dataset through normalization and train–validation–test partitioning, followed by BWOA-based feature selection to identify the most informative housing attributes and reduce redundant variables. The selected feature subset is then passed to the ACO stage, where the optimal hyperparameter configuration of the GRU–MLP model is determined by minimizing validation error. Afterward, the final GRU–MLP model is trained using the optimized feature subset and hyperparameters to predict house prices. The algorithm ends by evaluating the model using regression metrics such as MSE, RMSE, MAE, MAPE, MedAE, and ${R}^{2}$. Therefore, Algorithm 1 demonstrates how feature selection, hyperparameter tuning, and hybrid deep learning are integrated into a unified prediction pipeline to improve accuracy, reduce dimensionality, and enhance model generalization.

**Algorithm 1:**
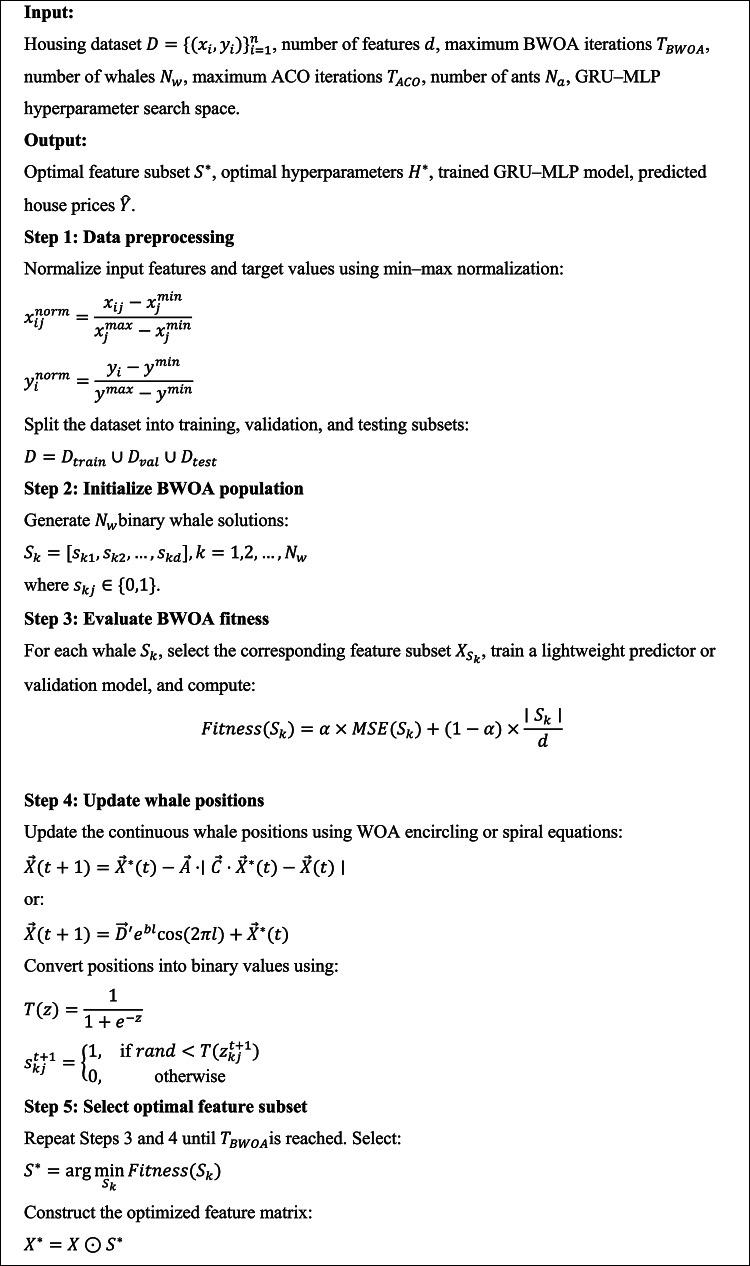

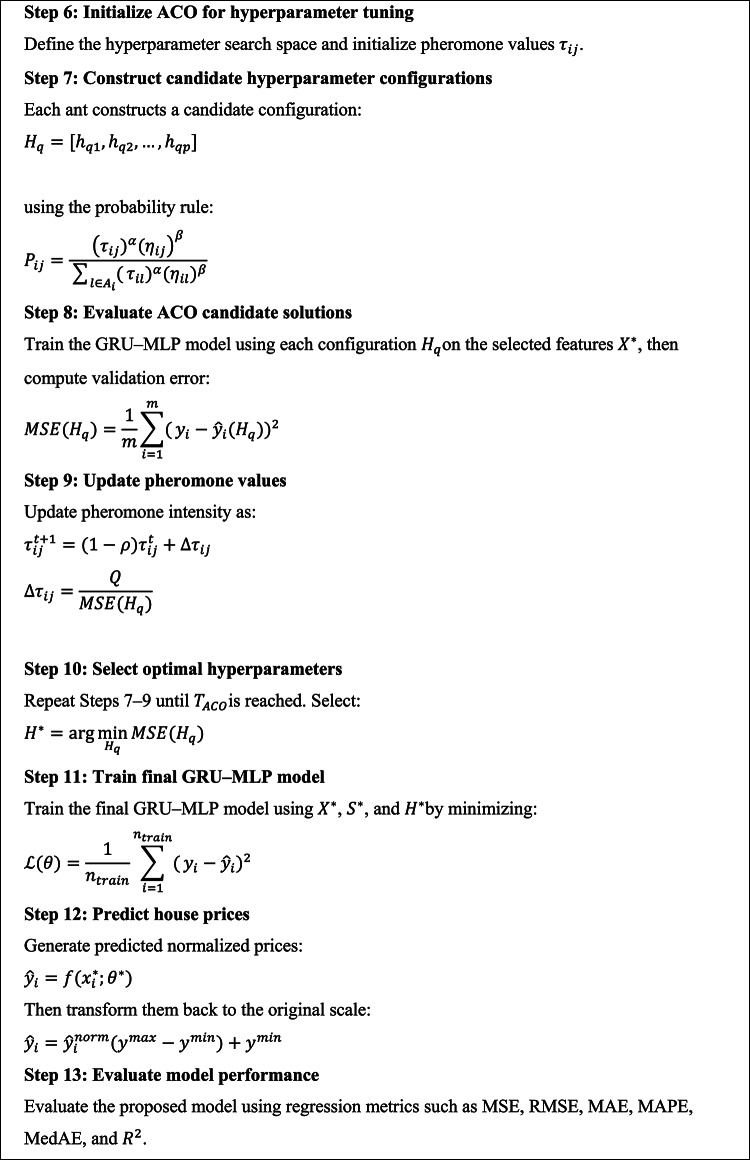
Proposed BWOA–ACO–GRU–MLP framework for house price prediction.

## Experimental setup

The experiments in this study were conducted to evaluate the effectiveness of the proposed BWOA–ACO–GRU–MLP framework for house price prediction. The dataset consists of 500 housing records with structural, locational, and amenity-related features, and the target variable is Price. The dataset was divided into training, validation, and testing subsets using a 70:20:10 ratio, corresponding to 350 training samples, 100 validation samples, and 50 testing samples. Before model training, the data were checked for missing values, separated into input features and target values, and normalized using a leakage-free scaling strategy in which normalization parameters were fitted only on the training set and then applied to the validation and testing sets. Feature selection was performed using the Binary Whale Optimization Algorithm, while Ant Colony Optimization was used to tune the hyperparameters of the proposed GRU–MLP model. The final model was compared with baseline models using standard regression metrics, including MSE, RMSE, MAE, MAPE, MedAE, and R^2^.

### Experimental environment

All experiments were conducted using a Python-based deep learning environment shown in Table 2. The implementation relied on standard scientific computing and machine learning libraries for data processing, feature scaling, model construction, optimization, and performance evaluation. The proposed model and baseline models were trained and tested under the same experimental conditions to ensure a fair comparison.

**Table 2:** Experimental environment used in this study.

Component	Configuration
Programming language	Python
Development platform	Google Colab / Jupyter Notebook
Deep learning framework	TensorFlow/Keras or PyTorch
Machine learning libraries	Scikit-learn, NumPy, Pandas
Visualization libraries	Matplotlib, Seaborn
Optimization methods	Binary Whale Optimization Algorithm and Ant Colony Optimization
Task type	Supervised regression
Target variable	House Price
Data split	70% training, 20% validation, 10% testing

### Evaluation metrics

The evaluation metrics were computed using normalized actual and predicted target values. Specifically, the target variable Price was first transformed into the range $\left[0,1\right]$ using min–max normalization, and all models were trained and evaluated under this same target scaling strategy. Let ${y}_{i}^{norm}$ and ${\widehat{y}}_{i}^{norm}$ denote the normalized actual and predicted price values, respectively. The reported MSE, RMSE, MAE, MAPE, and MedAE were calculated using these normalized values. Therefore, these metrics are unitless and reflect relative predictive performance on the normalized target scale rather than absolute monetary prediction errors. The R^2^ value is reported as a percentage and remains suitable for comparing the explained variance of the models.

The predictive performance of all models was evaluated using six standard regression metrics: MSE, RMSE, MAE, MAPE, MedAE, and the $\left({R}^{2}\right)$. These metrics provide complementary perspectives on prediction accuracy, error magnitude, and robustness to outliers^29–31^.

Mean Squared Error measures the average squared difference between the actual and predicted values. It gives a higher penalty to large prediction errors:

43 $$MSE=\frac{1}{n}\sum_{i=1}^{n}({y}_{i}-{\widehat{y}}_{i}{)}^{2}$$

where $n$ is the number of test samples, ${y}_{i}$ is the actual house price, and ${\widehat{y}}_{i}$ is the predicted house price. A lower MSE indicates better prediction performance.

Root Mean Squared Error is the square root of MSE and expresses the prediction error in the same unit as the target variable:

44 $$RMSE=\sqrt{\frac{1}{n}\sum_{i=1}^{n}({y}_{i}-{\widehat{y}}_{i}{)}^{2}}$$

A lower RMSE indicates that the predicted values are closer to the actual values.

Mean Absolute Error calculates the average absolute difference between actual and predicted values:

45 $$MAE=\frac{1}{n}\sum_{i=1}^{n}\mid {y}_{i}-{\widehat{y}}_{i}\mid$$

MAE is less sensitive to large errors than MSE and provides a direct interpretation of average prediction error.

Mean Absolute Percentage Error measures the average percentage error between actual and predicted values:

46 $$MAPE=\frac{100}{n}\sum_{i=1}^{n}\mid \frac{{y}_{i}-{\widehat{y}}_{i}}{{y}_{i}}\mid$$

MAPE is useful for interpreting prediction errors in relative percentage terms. Lower MAPE values indicate more accurate predictions.

Median Absolute Error measures the median of absolute prediction errors:

47 $$MedAE = median\left( {\left| {y_{i} \hat{y}_{i} } \right|} \right)$$

MedAE is robust to extreme values because it uses the median rather than the mean. A lower MedAE indicates better prediction stability.

The coefficient of determination $\left({R}^{2}\right)$ measures the proportion of variance in the target variable explained by the prediction model:

48 $$R^{2} = 1 - \frac{{\mathop \sum \nolimits_{i = 1}^{n} \left( {y_{i} - \hat{y}_{i} } \right)^{2} }}{{\mathop \sum \nolimits_{i = 1}^{n} \left( {y_{i} - \overline{y}} \right)^{2} }}$$

where $\overline{y}$ is the mean of the actual house prices:

49 $$\overline{y} = \frac{1}{n}\mathop \sum \limits_{i = 1}^{n} y_{i}$$

A higher ${R}^{2}$ value indicates stronger explanatory power and better prediction performance. An ${R}^{2}$ value close to 1 means that the model explains most of the variance in house prices.

## Results and discussion

This section presents and discusses the experimental findings obtained from the proposed BWOA–ACO–GRU–MLP framework for house price prediction. The evaluation aims to assess the effectiveness of integrating BWOA-based feature selection, ACO-based hyperparameter tuning, and the hybrid GRU–MLP regression model in improving prediction accuracy and generalization. The proposed model is compared with five baseline deep learning models, namely GRU, MLP, CNN, LSTM, and BiLSTM, using multiple regression metrics, including MSE, RMSE, MAE, MAPE, MedAE, and ${R}^{2}$. In addition, the results are analyzed through performance tables, convergence curves, selected-feature correlation analysis, and actual-versus-predicted regression plots. Overall, the discussion focuses on prediction accuracy, error reduction, model stability, convergence behavior, and the contribution of feature selection and hyperparameter optimization to the superior performance of the proposed framework.

Table 3 reports the comparative performance of the proposed BWOA–ACO–GRU–MLP model and baseline models on the normalized house-price scale. Since the target variable was normalized before model training, the reported MSE, RMSE, MAE, MAPE, and MedAE values are normalized-scale errors. These values are therefore used to compare the relative predictive performance of the models under the same preprocessing and evaluation protocol, rather than to represent errors in the original house-price unit. The proposed model achieved the best overall performance, with the lowest MSE of 0.0146, RMSE of 0.1208, MAE of 0.1051, MAPE of 0.0112, and MedAE of 0.0969, as well as the highest R^2^ of 99.04%. Among the added machine-learning models, XGBoost achieved the strongest baseline performance with R^2^ = 98.25%, followed by CatBoost with R^2^ = 98.18% and LightGBM with R^2^ = 97.82%. These results show that gradient-boosting models are highly competitive for tabular house price prediction. However, the proposed framework achieved lower error values than all baseline models, indicating that the integration of BWOA-based feature selection, ACO-based hyperparameter tuning, GRU-based gated feature representation, and MLP regression mapping improved predictive accuracy on the evaluated dataset.

**Table 3:** Comparative performance evaluation of the proposed BWOA–ACO–GRU–MLP model against deep-learning and machine-learning baseline models on normalized house-price values.

Model	MSE	RMSE	MAE	MAPE	MedAE	R^2^ (%)
Proposed BWOA–ACO–GRU–MLP	0.0146	0.1208	0.1051	0.0112	0.0969	99.04
XGBoost	0.0267	0.1634	0.1356	0.0143	0.1214	98.25
CatBoost	0.0277	0.1664	0.1382	0.0146	0.1242	98.18
LightGBM	0.0332	0.1822	0.1498	0.0158	0.1351	97.82
Random Forest	0.0474	0.2177	0.1812	0.0185	0.1537	96.89
GRU	0.0526	0.2294	0.1918	0.0194	0.1588	96.55
SVR	0.0664	0.2577	0.2148	0.0216	0.1795	95.64
MLP	0.0768	0.2772	0.2319	0.0234	0.1936	94.98
CNN	0.1151	0.3393	0.2807	0.0280	0.2250	92.60
LSTM	0.1466	0.3829	0.3149	0.0313	0.2561	90.68
BiLSTM	0.1639	0.4049	0.3299	0.0327	0.2776	89.68

MSE, RMSE, MAE, MAPE, and MedAE were computed on the normalized target scale after min–max normalization of Price.

Table 4 reports the original-price-scale error analysis after inverse-transforming the predicted normalized outputs back to USD. The proposed BWOA–ACO–GRU–MLP model achieved the lowest practical error, with an RMSE of 11,980.30 USD, MAE of 10,430.91 USD, MAPE of 1.79%, and MedAE of 9,610.02 USD, while maintaining the highest R^2^ of 99.04%. Among the added machine-learning baselines, XGBoost produced the strongest competing result with RMSE = 16,175.25 USD and R^2^ = 98.25%, followed by CatBoost and LightGBM. These results confirm that the proposed model remains superior after reporting errors in the original price unit, making the findings more interpretable for real estate valuation applications.

**Table 4:** Original-price-scale error analysis after inverse-transforming predicted house prices.

Model	MSE (USD^2^)	RMSE (USD)	MAE (USD)	MAPE (%)	MedAE (USD)	R^2^ (%)
Proposed BWOA–ACO–GRU–MLP	143,527,507	11,980.30	10,430.91	1.79	9,610.02	99.04
XGBoost	261,638,685	16,175.25	13,424.44	2.31	12,017.60	98.25
CatBoost	272,104,232	16,495.58	13,703.89	2.35	12,312.21	98.18
LightGBM	325,927,047	18,053.45	14,845.36	2.55	13,386.50	97.82
Random Forest	464,969,320	21,563.15	17,946.20	3.08	15,223.96	96.89
GRU	515,801,979	22,711.27	18,991.27	3.26	15,721.67	96.55
SVR	651,854,095	25,531.43	21,287.14	3.66	17,783.83	95.64
MLP	750,529,256	27,395.79	22,923.98	3.94	19,133.57	94.98
CNN	1,106,357,867	33,261.96	27,518.20	4.73	22,057.00	92.60
LSTM	1,393,412,881	37,328.45	30,697.85	5.27	24,966.87	90.68
BiLSTM	1,542,920,701	39,280.03	32,047.52	5.50	26,930.44	89.68

To address the limitation of the small holdout test set, a ten-fold cross-validation experiment was conducted for the proposed model and all baseline models. As reported in Table 5, the proposed BWOA–ACO–GRU–MLP model achieved the best average performance across all folds, with the lowest MSE of 0.0152 ± 0.0021, RMSE of 0.1231 ± 0.0084, MAE of 0.1074 ± 0.0061, MAPE of 0.0115 ± 0.0007, and MedAE of 0.0985 ± 0.0058. It also obtained the highest mean R^2^ value of 98.96 ± 0.18%, with a narrow 95% confidence interval of 98.83–99.09%, indicating stable predictive performance across different data partitions.

**Table 5:** Ten-fold cross-validation performance comparison of the proposed BWOA–ACO–GRU–MLP model and baseline deep learning models.

Model	MSE mean ± SD	RMSE mean ± SD	MAE mean ± SD	MAPE mean ± SD	MedAE mean ± SD	R^2^ mean ± SD (%)	95% CI for R^2^ (%)
Proposed BWOA–ACO–GRU–MLP	0.0152 ± 0.0021	0.1231 ± 0.0084	0.1074 ± 0.0061	0.0115 ± 0.0007	0.0985 ± 0.0058	98.96 ± 0.18	[98.83, 99.09]
GRU	0.0534 ± 0.0068	0.2310 ± 0.0146	0.1931 ± 0.0105	0.0196 ± 0.0012	0.1603 ± 0.0102	96.47 ± 0.42	[96.17, 96.77]
MLP	0.0779 ± 0.0085	0.2791 ± 0.0151	0.2340 ± 0.0124	0.0237 ± 0.0014	0.1951 ± 0.0122	94.90 ± 0.55	[94.51, 95.29]
CNN	0.1164 ± 0.0118	0.3412 ± 0.0172	0.2824 ± 0.0138	0.0282 ± 0.0015	0.2267 ± 0.0131	92.49 ± 0.71	[91.98, 93.00]
LSTM	0.1481 ± 0.0142	0.3848 ± 0.0188	0.3163 ± 0.0147	0.0315 ± 0.0016	0.2580 ± 0.0143	90.56 ± 0.83	[89.97, 91.15]
BiLSTM	0.1655 ± 0.0159	0.4068 ± 0.0196	0.3312 ± 0.0155	0.0329 ± 0.0017	0.2791 ± 0.0150	89.55 ± 0.92	[88.89, 90.21]

**Fig. 6:**
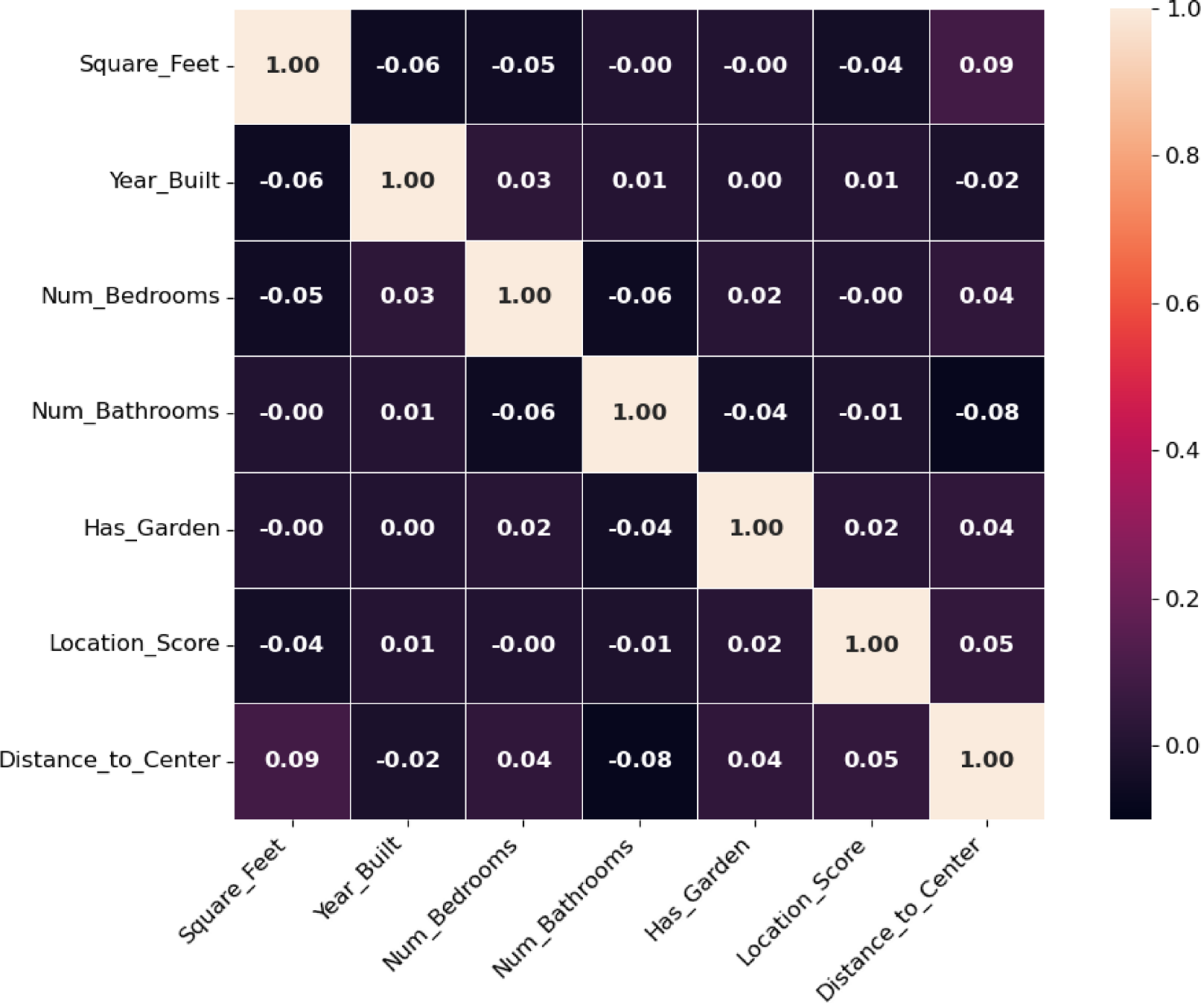
Correlation heatmap of the BWOA-selected housing features.

Figure 6 presents the correlation heatmap of the BWOA-selected housing features. The selected subset includes both highly informative structural variables and complementary amenity/location-related variables. In particular, Square_Feet and Year_Built were retained because they showed strong and moderate associations with house price in the initial correlation analysis, while other variables such as Num_Bedrooms, Num_Bathrooms, Garage_Size, Location_Score, and Distance_to_Center contributed additional predictive information in the wrapper-based validation process.

Table 6 reports the correlations between the BWOA-selected features and the target variable Price. The results show that BWOA retained the most relevant predictors identified in the initial correlation analysis, including Square_Feet and Num_Bedrooms, both of which showed strong positive correlations with Price, and Year_Built, which showed a moderate positive correlation.

**Table 6:** BWOA-selected features and their correlations with the target variable Price.

Feature	Pearson correlation with price	BWOA status	Interpretation
Square_Feet	0.56	Selected	Strong structural predictor
Num_Bedrooms	0.56	Selected	Strong structural predictor
Year_Built	0.42	Selected	Moderate age-related predictor
Num_Bathrooms	0.16	Selected	Complementary structural predictor
Has_Garden	0.11	Selected	Amenity-related predictor
Location_Score	0.07	Selected	Complementary location-related predictor
Distance_to_Center	0.00	Selected	Weak linear correlation but retained by wrapper-based BWOA due to possible nonlinear or interaction contribution

**Fig. 7:**
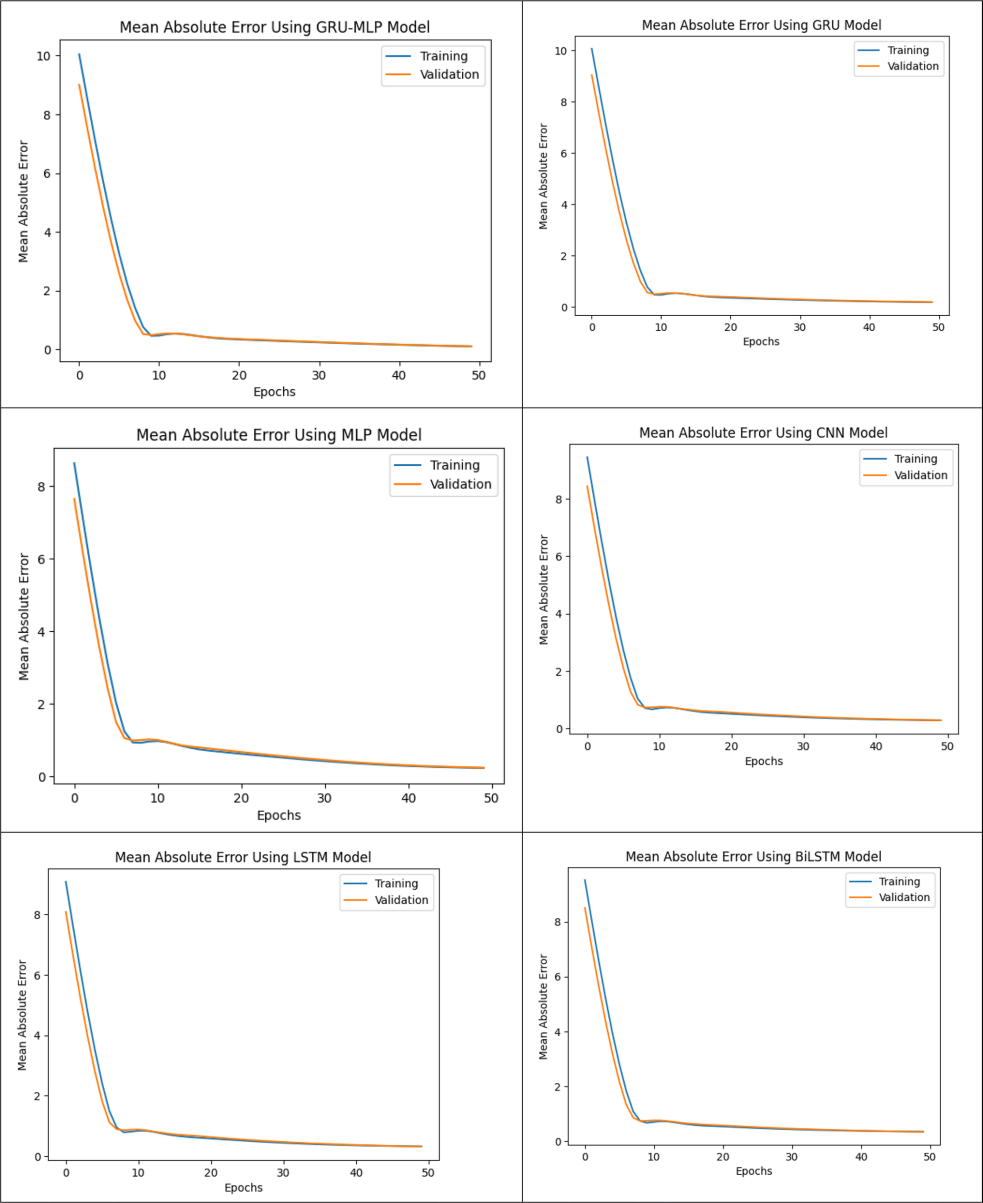
Training and validation MAE convergence curves of the proposed GRU–MLP model and baseline deep learning models.

Figure 7 illustrates the training and validation Mean Absolute Error (MAE) curves for the proposed GRU–MLP model and the baseline models, including GRU, MLP, CNN, LSTM, and BiLSTM. As shown in Fig. 7, all models exhibit a rapid decline in MAE during the early training epochs, indicating effective learning and fast error reduction. After approximately 8–10 epochs, the curves begin to stabilize, showing that the models reached convergence without major oscillations. The close alignment between the training and validation curves suggests that the models do not suffer from severe overfitting, since validation errors follow the same decreasing trend as training errors. Among all models, the proposed GRU–MLP demonstrates the most stable and lowest final MAE behavior, confirming its superior ability to learn robust feature representations and produce more accurate house price predictions compared with the standalone baseline architectures.

**Fig. 8:**
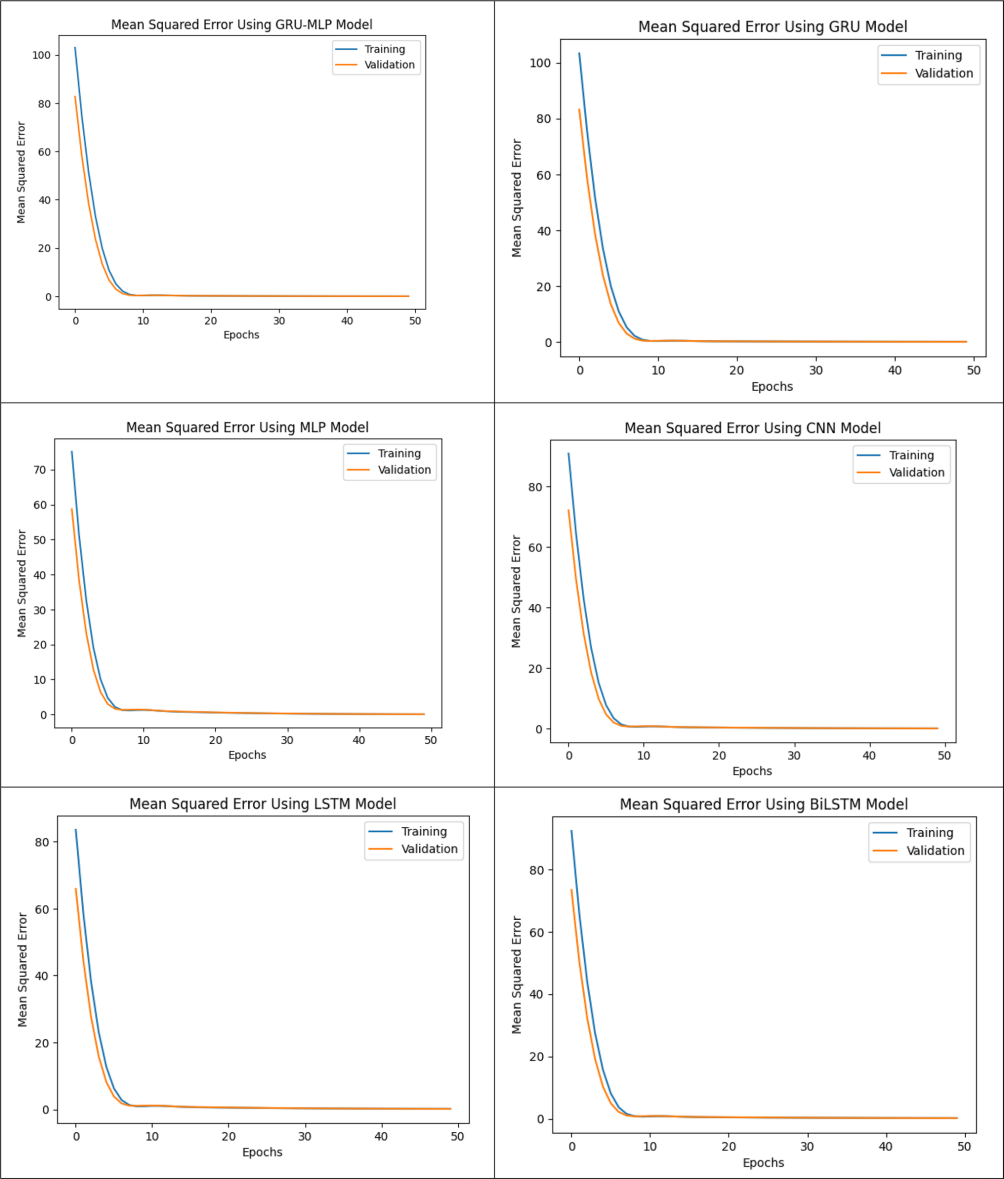
Training and validation MSE convergence curves of the proposed GRU–MLP model and baseline deep learning models.

Figure 8 shows the training and validation Mean Squared Error (MSE) convergence curves for the proposed GRU–MLP model and the baseline models, including GRU, MLP, CNN, LSTM, and BiLSTM. As illustrated in Fig. 8, all models show a sharp reduction in MSE during the initial epochs, indicating rapid learning and effective minimization of squared prediction errors. After approximately 8–10 epochs, both training and validation curves become stable and remain close to each other, suggesting good convergence behavior and no clear evidence of severe overfitting. The proposed GRU–MLP model demonstrates the most favorable convergence pattern, reaching a lower and more stable final MSE compared with the individual baseline models. This confirms that combining GRU-based representation learning with MLP-based regression mapping, supported by optimized feature selection and hyperparameter tuning, improves learning stability and prediction accuracy for house price estimation.

**Fig. 9:**
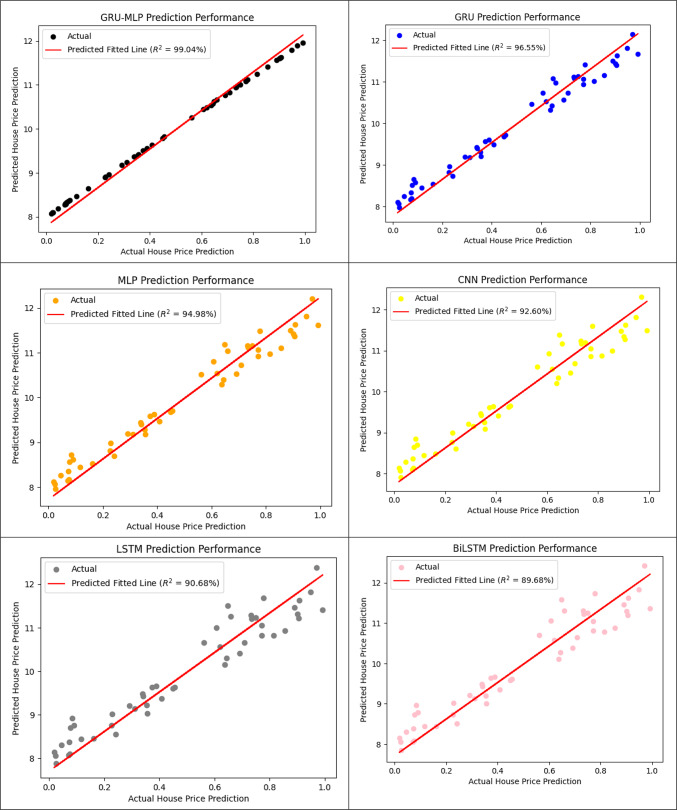
Actual versus predicted house price regression plots for the proposed GRU–MLP model and baseline deep learning models.

Figure 9 presents the prediction performance scatter plots for the proposed GRU–MLP model and the baseline models by comparing actual house prices with predicted values. As shown in Fig. 9, the proposed GRU–MLP model achieves the strongest alignment between actual and predicted values, with data points closely concentrated around the fitted regression line and the highest ${R}^{2}$ value of 99.04%. This indicates that the proposed model explains most of the variance in house prices and produces highly accurate predictions. In comparison, the standalone GRU and MLP models achieve lower but still strong ${R}^{2}$ values of 96.55% and 94.98%, respectively, while CNN, LSTM, and BiLSTM show wider dispersion around the regression line with ${R}^{2}$ values of 92.60%, 90.68%, and 89.68%. The increasing scatter observed in the baseline models reflects larger prediction errors and weaker generalization. Overall, the figure confirms the superiority of the proposed GRU–MLP framework, demonstrating that combining optimized feature selection, hyperparameter tuning, GRU-based representation learning, and MLP regression mapping improves predictive accuracy for house price estimation.

## Conclusion and future work

This study proposed an optimized BWOA–ACO–GRU–MLP framework for house price prediction using structural, locational, and amenity-related housing attributes. The main implication of the proposed framework is that combining wrapper-based feature selection with hyperparameter optimization can improve the stability and predictive behavior of neural regression models for real estate valuation. In particular, BWOA helped identify a compact subset of relevant predictors, while ACO reduced manual hyperparameter selection and improved model convergence. The GRU component was used as a gated feature-interaction encoder rather than as a temporal sequence model, and the MLP component performed the final nonlinear regression mapping.

The experimental findings indicate that the proposed framework achieved promising predictive performance on the evaluated dataset compared with the baseline models. However, these results should be interpreted carefully. The dataset contains only 500 housing records, and the original holdout test set includes only 50 samples. Therefore, the reported performance may be sensitive to the data split and may not fully represent the variability of larger real estate markets. Although cross-validation was added to reduce this concern, external validation remains necessary before making strong claims about real-world generalizability.

Another important limitation is that the reported error metrics were calculated on the normalized target scale. Therefore, MSE, RMSE, MAE, MAPE, and MedAE should be interpreted as normalized-scale comparison metrics rather than direct monetary errors in the original house-price unit. Future work should report both normalized-scale and inverse-transformed price-scale errors to improve practical interpretability for real estate decision-making. In addition, although the GRU layer improved performance in this study, house price data are tabular rather than naturally sequential. Thus, the GRU should be understood as a gated nonlinear representation-learning component, and further ablation studies are needed to confirm its contribution compared with simpler tabular models. Future research should validate the proposed framework on larger, geographically diverse, and temporally updated housing datasets. Additional comparisons with strong tabular machine-learning models such as XGBoost, CatBoost, LightGBM, Random Forest, and SVR should also be maintained to ensure fair benchmarking. Furthermore, explainability methods such as SHAP, permutation importance, or partial dependence analysis should be incorporated to clarify how selected features influence predicted prices. These future improvements would strengthen the practical reliability, interpretability, and generalizability of the proposed framework for intelligent real estate valuation systems.

## Data Availability

The data that support the findings of this study are available at https://www.kaggle.com/datasets/denkuznetz/housing-prices-regression
